# Transmembrane domain quality control systems operate at the endoplasmic reticulum and Golgi apparatus

**DOI:** 10.1371/journal.pone.0173924

**Published:** 2017-04-06

**Authors:** Kit Briant, Nicholas Johnson, Eileithyia Swanton

**Affiliations:** Division of Molecular and Cellular Function, School of Biological Sciences, Faculty of Biology, Medicine and Health, University of Manchester, Manchester Academic Health Science Centre, Manchester, United Kingdom; University of Pittsburgh, UNITED STATES

## Abstract

Multiple protein quality control systems operate to ensure that misfolded proteins are efficiently cleared from the cell. While quality control systems that assess the folding status of soluble domains have been extensively studied, transmembrane domain (TMD) quality control mechanisms are poorly understood. Here, we have used chimeras based on the type I plasma membrane protein CD8 in which the endogenous TMD was substituted with transmembrane sequences derived from different polytopic membrane proteins as a mode to investigate the quality control of unassembled TMDs along the secretory pathway. We find that the three TMDs examined prevent trafficking of CD8 to the cell surface via potentially distinct mechanisms. CD8 containing two distinct non-native transmembrane sequences escape the ER and are subsequently retrieved from the Golgi, possibly via Rer1, leading to ER localisation at steady state. A third chimera, containing an altered transmembrane domain, was predominantly localised to the Golgi at steady state, indicating the existence of an additional quality control checkpoint that identifies non-native transmembrane domains that have escaped ER retention and retrieval. Preliminary experiments indicate that protein retained by quality control mechanisms at the Golgi are targeted to lysosomes for degradation.

## Introduction

Between 20% and 30% of the proteome of eukaryotic organisms is predicted to be integral membrane proteins [1]. These proteins are typically synthesised at the endoplasmic reticulum (ER), where transmembrane domains (TMDs) are integrated into the lipid bilayer and cytoplasmic/luminal domains undergo the folding steps and post-translational modifications necessary to achieve the native structure [2]. From the ER, correctly folded proteins are incorporated into transport vesicles for delivery to the cis-Golgi and subsequently to the trans-Golgi network (TGN), from where they are sorted to their site of function [3]. In contrast, proteins that fail to attain the correct conformation are prevented from moving along the secretory pathway by a series of quality control checkpoints, including those at the ER, Golgi apparatus and plasma membrane [4–6].

The best studied quality control checkpoint is the ER system, which identifies non-native features of proteins that have failed to fold correctly and retains them in the ER. Retained proteins may undergo further rounds of folding, or be transferred to the ER-associated degradation (ERAD) machinery which initiates ubiqutination and movement of the misfolded protein back across the ER membrane into the cytosol for degradation by the proteasome [7]. The molecular mechanisms underlying the quality control of cytoplasmic and luminal domains at the ER are relatively well understood and involve the recognition of non-native determinants, such as exposed hydrophobic patches, by molecular chaperones [8, 9]. In addition to cytoplasmic and luminal domains, integral membrane proteins also contain a variable number of hydrophobic TMDs composed of 18–25 amino acids that form α-helices which span the membrane to integrate the protein into the bilayer. TMDs are not simply inert membrane anchors, but typically possess specific properties that contribute to the structure, function and location of the native protein [10, 11]. The intra- and intermolecular assembly of TMDs is a critical step in the biogenesis of many membrane proteins [11–13]. However, relatively little is known about how and where proteins containing TMDs which have failed to fold or assemble correctly are identified along the secretory pathway. A number of ER quality control factors have been shown to contribute to the recognition and ERAD targeting of integral membrane proteins possessing TMD defects, including the lectin calnexin [14–17], E3 ubiquitin ligases [18, 19], RHBDL4 [20] and UGGT [21]. Mechanistically, recognition of aberrant TMDs may be mediated directly by the membrane spanning regions of these quality control proteins, as was elegantly shown for the yeast E3 ligase Hrd1p [18]. Although the exact nature / structure of the TMD defects they recognise are not clear, a common idea is that misfolding or misassembly of TMDs causes exposure of non-native features such as polar residues or mismatched hydrophobic lengths that would normally be shielded from the bilayer in the native structure [13, 22, 23]. Indeed, charged or strongly polar intramembrane residues cause ER retention of unassembled subunits of several oligomeric membrane proteins, and can also induce retention and ERAD of reporter proteins [24–27]. However, some membrane proteins with unassembled TMDs [28–30] or inferred TMD defects [31–33] are able to exit the ER. In some cases, unassembled subunits of oligomeric membrane proteins that leave the ER may be retrieved by Rer1, a sorting receptor localised to the cis-Golgi that recognises exposed polar residues in the TMDs of cargo proteins and returns them to the ER [28, 30, 34, 35]. Rer1 has also recently been implicated in the ER retention of misfolded membrane proteins in mammalian cells [14, 36], suggesting it may have a more general role in the identification of defective TMDs in the early secretory pathway.

In addition to quality control at the ER, some non-native membrane proteins have been shown to be retained in the Golgi of mammalian cells and targeted to lysosomes for degradation, suggesting additional quality control checkpoints in this compartment [31, 37]. Most known substrates of the Golgi quality control pathway to date have been characterised in yeast, where recognition is accompanied by sorting into intraluminal vesicles in multivesicular bodies and degradation in the vacuole [6, 38, 39]. In yeast, the presence of polar residues within TMDs is important for sorting of vacuolar proteins at the Golgi [40–42], and has also been proposed to contribute to the selection of quality control substrates to be targeted to the vacuole for degradation [40]. Likewise, in mammalian cells, introduction of two charged residues into the TMD of hemagglutanin resulted in targeting to the lysosome for degradation, indicating TMDs that escape ER quality control (ERQC) are subject to quality control at the Golgi [35]. However, few mammalian Golgi quality control substrates have been characterised and it is not known if misassembled or aberrant TMDs can be directly recognised in this organelle.

The objectives of this study were to investigate where and how unassembled TMDs are identified within the secretory pathway of mammalian cells. Using chimeric reporters based on the single spanning membrane protein CD8, we show that TMD sequences derived from different polytopic membrane proteins prevent transport to the cell surface and cause ER localisation. ER localisation is achieved through a combination of retention and retrieval mechanisms, and we provide evidence that Rer1 contributes to retrieval. Shortening one of these TMD sequences allowed the chimera to escape from the ER, but resulted in Golgi localisation, suggesting a second checkpoint at the Golgi may recognise non-native TMDs.

## Materials and methods

### Antibodies and reagents

α-CD8 and rabbit α-HA were obtained from Sigma, α-BAP31, α-Actin and α-tubulin from AbCam, α-ERGIC53 from Alexis, mouse α-HA from Santa Cruz, α-EEA1 from Cell signalling and α-GM130 from BD biosciences. IRDye 800 CW and IRDye 680 RD antibodies were from LI-COR, and fluorescently conjugated secondary antibodies for microscopy were from Jackson Laboratories (Stratech Scientific). The inhibitors leupeptin (Enzo Life Sciences), pepstatin A (Sigma), Z-LLF-CHO (PSII, Calbiochem), and cycloheximide (CHX, Sigma) were used at a final concentration of 0.5 mM, 1 μg/ml, 10 μM, and 100 μg/ml respectively.

### Constructs

CD8^TMD*^ and CD8^*WT*^ were generated as described previously [43]. CD8^*TMD23*^ was generated using the deletion protocol from the Phusion Site-directed mutagenesis kit (Thermo Scientific). CD8^*SERCA*^ and CD8^*PMCA*^ cDNAs [44] were provided by Dr J.M. East, University of Southampton. These constructs contained TMD10 from rat SERCA and rabbit PMCA, and were cloned into the pcDNA5.1/FRT/TO vector with a HA tag added at the C-terminus by PCR. It should be noted that CD8^*WT*^ and CD8^TMD*^ differed from CD8^*PMCA*^ and CD8^*SERCA*^ in the 4 residues predicted to be immediately adjacent to the TMD on the cytoplasmic face of the membrane (NHRN compared with KRLK respectively; S1A Fig), although both sequences would be expected to ensure the same topology according to the positive inside rule [45]. All further constructs were generated by site directed mutagenesis. All sequences were verified by DNA sequencing (GATC Biotech).

### Transfections

Stable HeLa TRex Flp-In cells expressing CD8 chimeras were generated by transfecting parental TRex HeLa cells (provided by Steven Taylor, University of Manchester) using Lipofectamine LTX (Invitrogen) following manufacturer’s instructions. Transfected cells were selected using 100 μg/ml hygromycin B (ForMedium) and 4 μg/ml blasticidin (InvivoGen). Once established, cell lines were maintained in complete DMEM (DMEM (Sigma) supplemented with 10% FBS, 2 mM L-glutamine and 1% non-essential amino acids) at 37°C, 8% CO_2_. All experiments were performed after inducing expression with 1 μg/ml tetracycline for 16–20 hours unless otherwise stated. For siRNA transfections, cells were seeded at 25,000 cells / well in a 12 well dish. The following day, cells were transfected with INTERFERin (Polyplus Transfection), using a final concentration of 20 nM siRNA duplex. Experiments were performed 48–72 hours post-transfection. Rer1 targeting siRNA oligo 1 sequence: 5’-UAUCAGUCCUGGCUAGAC-3’; Rer1 siRNA oligo 2 sequence: 5’-UGCGAGUUACAGAAUGUCUGA-3’. Calnexin siRNA sequence: 5’-UCAUCAUCGGUAUCGUCUU-3’

### Quantitative PCR

RNA was extracted from siRNA treated cells 72 hours post-transfection using TRIzol (Invitrogen). 1μg RNA was converted to cDNA using the AMV first strand cDNA synthesis kit for RT-PCR (Roche) and subsequently diluted at 3 5-fold dilutions. qPCR was carried out using SYBR green mastermix (Eurogentec) and an Opticon qPCR thermal cycler (Genetic Research Instrumentation). Rer1 transcript levels were normalised relative to GAPDH. Rer1 primers were from QIAgen, GAPDH primers were Fwd: AAGGGCATCCTGGGCTAC and Rev: GTGGAGGAGTGGGTGTCG.

### Microscopy

For colocalisation analysis, cells were induced to express the indicated CD8 chimeras overnight. Subsequently cells were fixed in 3% formaldehyde (Sigma) in PBS (137 mM NaCl; 2.7 mM KCl; 10 mM Na_2_HPO_4_; 2 mM KH_2_PO_4_; pH 7.4) for 15 min at room temperature. Unreacted formaldehyde was quenched with glycine. Cells were permeabilised with 0.1% Triton X-100 for 4 min. For selective permeabilisation (S2 Fig), cells were either permeabilised with 0.1% Triton X-100 for 4 min or with 40μg/ml digitonin for 4 min. CD8 chimeras and subcellular markers were labelled with primary antibodies for 30–60 min followed by the appropriate fluorescently conjugated secondary antibody. Cells were mounted in ProLong Gold Antifade Reagent with DAPI (Molecular Probes). For cell surface labelling, cells were pre-chilled for 20 min and labelled with α-CD8 antibodies for 30 min, all on iced water. Subsequently, cells were washed in cool PBS, fixed in formaldehyde and processed as above. For temperature block analysis, cells were incubated in CO_2_-independent media at 15°C or 37°C for 3 hours prior to fixation and processing as above. All images were captured using an Olympus BX-60 upright microscope with a 60X 1.40 N.A. PlanApo objective and a CoolSNAP EZ camera (Photometrics) using MetaMorph software (MDS Analytical Technologies). Image montages were created in ImageJ (http://rsbweb.nih.gov/ij/) and final figures collated in Illustrator CS5 (Adobe).

### SDS-PAGE and immunoblotting

Prior to SDS-PAGE, samples were heated in SDS-PAGE sample buffer (30 mM Tris pH 7.6; 2% SDS; 5% glycerol; 0.01% bromophenol blue; 100 mM DTT). Samples were separated on 12% acrylamide gels after heating. For immunoblot analysis, proteins were transferred onto nitrocellulose membrane (LI-COR) using a TE22 wet transfer tank (Hoefer). Immunoreactive bands were detected with the ODYSSEY Sa infrared imaging system and quantified using ODYSSEY Sa Infrared Imaging System Application Software Version 1.0.12 (LI-COR).

### Pulse labelling and maturation

Cells were starved in DMEM lacking met / cys (GIBCO) for 30 min at 37°C prior to labelling in the presence of 22 μCi/ml [^35^S] met / cys EasyTag EXPRESS35S protein labelling mix (PerkinElmer) at 37°C for 10 min. Subsequently, cells were washed in PBS and incubated in complete DMEM supplemented with 10 mM non-radioactive met / cys for the indicated chase time at 37°C. Cells were then washed in PBS and lysates were prepared by solubilisation in IP-Tx buffer (10 mM Tris-HCL pH 7.6, 140 mM NaCl, 1 mM EDTA, 1% (v/v) Triton X-100, 1 mM PMSF) followed by centrifugation at 15,000 g for 10 min at 4°C. Post-nuclear supernatants (PNS) were taken and incubated with α-HA antibody overnight at 4°C, prior to the addition of protein A sepharose (Genscript) at 4°C for a further 3 hours. Immuno-sepharose complexes were washed 3 times in IP-Tx buffer before resuspension in reducing sample buffer for analysis by SDS-PAGE and phosphorimaging.

### Trypsinisation of cell surface proteins

Cells were induced to express the indicated CD8 chimeras overnight. Subsequently, cells were either harvested directly in sample buffer (no trypsin) or incubated in 0.5% trypsin-EDTA solution (Sigma) at 37°C for 3 min to detach cells before incubating on slush ice for 90 min. Subsequently, cells were treated with 0.5 mg/ml soybean trypsin inhibitor (SBTI) for 15 min, washed, centrifuged and harvested in sample buffer for analysis by SDS-PAGE and immunoblotting.

### Biotinylation of cell surface proteins

Cells were induced to express the indicated CD8 chimeras overnight. Subsequently, cells were pre-chilled on ice and labelled with 0.5 mg/ml EZ-Link Sulfo-NHS-SS-biotin (Pierce Biotechnology) in PBS/CM (PBS + 0.9 mM CaCl_2_, 0.33 mM MgCl_2_) for 30 minutes with gentle rocking. Cells were washed with PBS/CM, and unreacted biotin was quenched with 50 mM NH_4_Cl in PBS/CM. Subsequently, cells were lysed in biotin lysis buffer (50 mM Tris pH 7.4, 150 mM NaCl, 5 mM EDTA, 1.25% TritonX-100, 0.25% SDS, 1 mM PMSF) followed by centrifugation at low speed for 15 min. Post-nuclear supernatants were incubated with NeutrAvidin beads (Thermo Scientific) for 2.5 h, washed 3 times in biotin lysis buffer and the resulting beads resuspended in reducing sample buffer for analysis by SDS-PAGE and immunoblotting.

### Cycloheximide chase analysis

Cells were induced to express the indicated CD8 chimeras overnight. Subsequently, cells were incubated at 37°C in complete DMEM containing 100 μg/ml cycloheximide to block further protein synthesis. Where indicated, inhibitors were added to the media throughout the chase. At the indicated time after the addition of CHX, cells were washed twice with PBS and lysed in sample buffer. All samples were analysed by SDS-PAGE and immunoblotting.

### Statistical analysis

Unpaired t-tests and one-way ANOVAs with Tukey’s multiple comparisons test were carried out where indicated using GraphPad Prism 7. * indicates p < 0.05, ** indicates p < 0.01.

## Results

### Diverse unassembled transmembrane domains cause ER localisation

**Fig 1:**
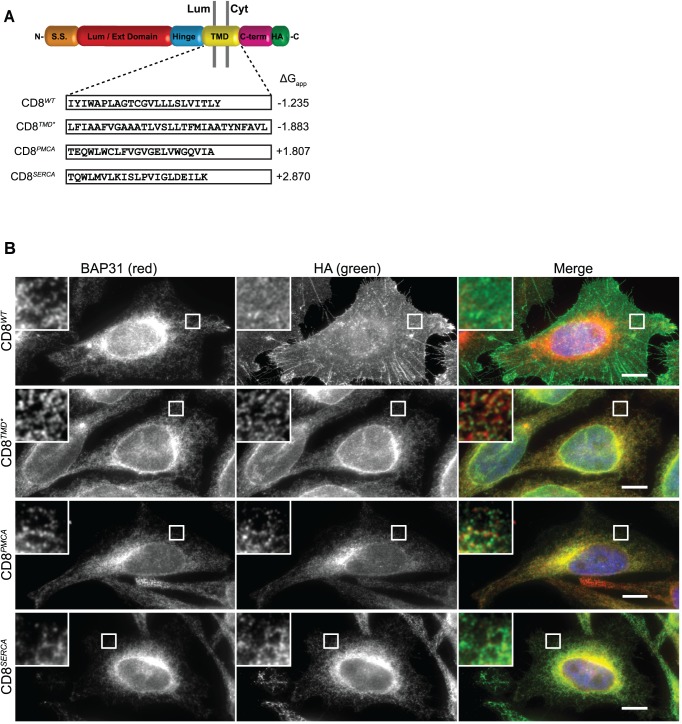
Diverse unassembled TMDs mediate ER localisation. (A) Cartoon schematic of the domain structure of CD8α. The TMD amino acid sequence and predicted ΔG_app_ for TMD membrane insertion of CD8^*WT*^, CD8^TMD*^, CD8^*PMCA*^ and CD8^*SERCA*^ are shown. (B) Cells expressing CD8^*WT*^, CD8^TMD*^, CD8^*PMCA*^ or CD8^*SERCA*^ were fixed with formaldehyde, permeabilised with Triton X-100 and co-immunostained with antibodies against BAP31 and the epitope tag HA. Cell nuclei were stained with DAPI (represented in blue in merged images). Scale bars indicate 10 μm.

We recently established a chimeric model protein for studying TMD quality control, in which the endogenous TMD of the type I membrane protein CD8 was replaced with TMD 4 of a 4-pass membrane protein (proteolipid protein, PLP; [43]). We showed that although the extracellular/luminal domain is folded, this chimera, termed CD8^TMD*^, is localised to the ER and degraded via ERAD [43]. Thus when expressed in the absence of TMDs 1–3 of PLP, this TMD sequence exposes signals that can be recognised by the ER quality control machinery. In order to examine whether TMD sequences from other multi-spanning membrane proteins also cause retention at the ER, we generated two further CD8 chimeras, in which the endogenous TMD was replaced with TMD 10 from two Ca^2+^-ATPases, PMCA3 and SERCA1, herein referred to as CD8^*PMCA*^ and CD8^*SERCA*^ respectively (Fig 1A). An HA epitope was fused to the end of the cytoplasmic C-terminus. These TMDs were chosen as, in contrast to the sequence present in CD8^TMD*^, they both possess a positive predicted ΔG_app_ for membrane insertion [46] (Fig 1A), allowing us to investigate marginally hydrophobic TMDs. This is important as previous work suggests that such TMDs may fail to integrate stably into the bilayer in the absence of partner TMDs, leading to their recognition by luminal ERQC [47, 48]. In addition, CD8^*PMCA*^ and CD8^*SERCA*^ differ substantially from CD8^TMD*^ in terms of their amino acid composition and length, and thus represent diverse transmembrane spanning sequences for comparison of quality control of unassembled TMDs.

Initially we examined the localisation of these constructs. In order to overcome potential problems associated with variable expression levels obtained by transient transfection, we generated HeLa cell lines stably expressing these CD8 chimeras under a tetracycline-inducible promoter. At steady state, CD8^*WT*^ was present primarily at the cell surface and showed little co-localisation with the ER-marker BAP31 (Fig 1B, top). In contrast, CD8^TMD*^ displayed a reticular staining pattern which overlapped with that of BAP31, indicating ER localisation (Fig 1B) as previously established [43]. Likewise, both CD8^*PMCA*^ and CD8^*SERCA*^ were observed in a reticular pattern typical of the mammalian ER and exhibited a high degree of co-localisation with BAP31 (Fig 1B), showing that these TMDs also caused localisation at the ER.

Given that the TMDs of CD8^*PMCA*^ and CD8^*SERCA*^ both had positive predicted ΔG_app_ values, indicating unfavourable energetics for membrane insertion, it was important to examine whether they were in fact membrane integrated. Marginally hydrophobic TMDs may fail to integrate stably into the ER membrane and thus translocate fully into the ER lumen in the absence of partner subunits [47, 48]. To this end, the accessibility of the extracellular/ER luminal CD8 domain and the HA epitope at the C-terminus of these chimeras was examined after selective permeabilisation. In cells treated with digitonin the membranes of the ER remained intact and the luminal lectin calreticulin was not accessible for antibody labelling (S2A Fig, bottom). Under these conditions, the HA epitope of CD8^TMD*^ and CD8^*PMCA*^ could be clearly detected (S2B and S2C Fig, bottom), indicating the C-termini of these proteins were correctly located on the cytoplasmic side of the ER. In contrast, labelling with the anti-CD8 antibody was only apparent after ER membranes had been permeabilised with Triton X-100 and was not detected in digitonin-permeabilised cells (S2B and S2C Fig), suggesting that the N-terminal domains of these proteins were located within the ER lumen. On the basis of these results, we conclude that CD8^TMD*^ and CD8^*PMCA*^ are integrated into the ER membrane with the correct topology. For CD8^*SERCA*^ however, neither the N- nor C-terminal domains were accessible to antibody labelling in digitonin-treated cells, but were revealed upon permeabilisation of ER membranes with Triton-X 100 (S2D Fig). This indicates that both the N- and C-termini of CD8^*SERCA*^ were located inside the ER lumen. Hence, this chimera may not be stably integrated into the ER membrane, but translocated fully into the ER lumen, as observed for other proteins containing marginally hydrophobic TMDs [47, 48]. Alkaline extraction experiments demonstrated that a large proportion of CD8^*SERCA*^ could be removed from cellular membranes upon treatment with sodium carbonate pH 11 (S3C Fig), confirming that this chimera is not membrane integrated. This is in contrast to CD8^TMD*^ [43] and CD8^*PMCA*^ (S3B Fig), which were almost completely resistant to carbonate extraction, remaining stably associated with the membrane fraction. Thus, whilst chimeras containing the three different non-native TMD sequences were localised at the ER, this may be achieved via distinct mechanisms.

### Retention and retrieval mechanisms contribute to ER localisation of unassembled transmembrane domains

**Fig 2:**
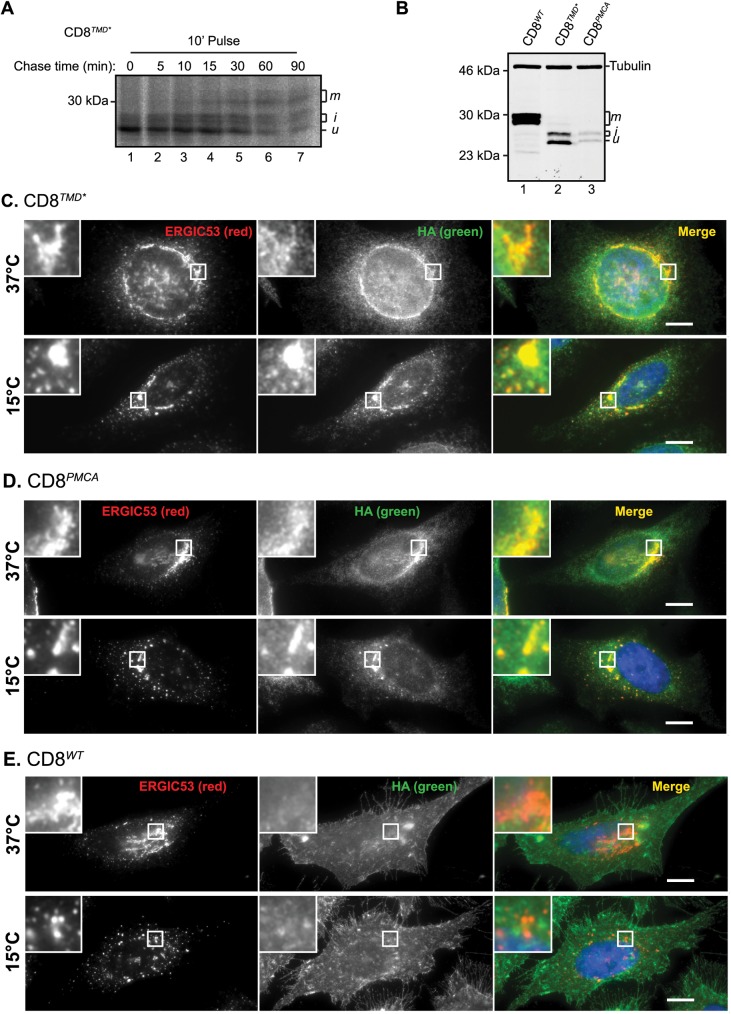
CD8^TMD*^ and CD8^*PMCA*^ are retrieved from the Golgi. (A) Cells expressing CD8^TMD*^ were pulse labelled with [^35^S] met/cys for 10 minutes and chased for up to 90 minutes as indicated. Samples were immunoprecipitated with antibodies against HA, separated by SDS-PAGE and analysed by phosphorimaging. (B) Whole cell lysates of cells expressing CD8^*WT*^, CD8^TMD*^ or CD8^*PMCA*^ were separated by SDS-PAGE and analysed by immunoblotting with antibodies against HA and tubulin. (C-E) Cells expressing (C) CD8^TMD*^, (D) CD8^*PMCA*^ or (E) CD8^*WT*^ were incubated at 15°C or 37°C for 3 hours prior fixation. Cells were co-immunostained with antibodies against ERGIC53 and the epitope tag HA. Scale bars indicate 10 μm.

The observation that CD8^TMD*^ and CD8^*PMCA*^ are membrane integrated and localised to the ER suggests that both of these TMD segments expose specific signals within the lipid bilayer that result in recognition by ERQC mechanisms. Such signals may be masked in the context of the full length proteins, since the TMDs from multispanning membrane proteins typically interact and/or assemble with neighbouring TMDs in their native conformation, as has been proposed for PLP [49]. Since little is known about TMD-based QC, we next examined the mechanisms underlying ER localisation of these chimeras. Whilst some unassembled or misfolded proteins are retained statically in the ER, others exit the ER and undergo retrieval from the Golgi [14, 28, 29, 36]. Our previous work showed that a proportion of CD8^TMD*^ receives Golgi post-translational modifications, suggesting that it is able to partially escape from the ER to later stages of the secretory pathway [43]. Thus, CD8^TMD*^ was initially synthesised as a 25 kDa species (Fig 2A, lane 1), respesenting the unmodified precursor (termed ‘u’) following pulse-labelling with [^35^S] amino acids [43, 50–52]. Over time, a proportion of the precursor was converted to several higher molecular weight forms (Fig 2A, lanes 3–7): two closely migrating bands of around 27 kDa representing intermediate O-glycoforms (termed ‘i’) previously shown to be generated in the cis-Golgi; and 2–3 species of approximately 30 kDa observed after 15 minutes of chase, representing more mature O-glycoforms (termed ‘m’) that are produced in the trans-Golgi network [43, 51, 52]. Consistent with the pulse-chase analysis, CD8^TMD*^ was predominantly observed in the unmodified and intermediate forms at steady state, with a small but detectable amount of the ‘m’ glycoforms (Fig 2B lane 2). In contrast, CD8^*WT*^ was observed almost entirely in the ‘m’ form (Fig 2B lane 1), consistent with its progression through the secretory pathway and localisation to the cell surface (Fig 1B). Like CD8^TMD*^, CD8^*PMCA*^ was also observed as a mixture of the unmodified precursor and the intermediate glycoforms at steady state (Fig 2B lane 3), showing that a proportion of this chimera also escapes the ER and reaches the Golgi apparatus to undergo O-glycosylation. Although the pattern of CD8^TMD*^ and CD8^*PMCA*^ glycoforms were comparable, their steady state expression level of CD8^*PMCA*^ appeared to be considerably lower than that of CD8^TMD*^ (Fig 2B, compare lanes 2 and 3). Since the expression vectors for the different chimeras are stably integrated into the same genomic locus in each cell line, the differences in intracellular levels of the proteins at steady state most likely reflect differences in the rates at which they are degraded rather than differences in transcription or translation efficiency.

Since CD8^TMD*^ and CD8^*PMCA*^ were predominantly localised to the ER at steady state (Fig 1B), these results indicate that chimeras which exit the ER are subsequently retrieved from the Golgi. In order to determine if this was the case, we examined the effect of reduced temperature on the subcellular distribution of the chimeras. Growth of cells at 15°C inhibits exit of proteins from the ER-Golgi intermediate compartment (ERGIC) in both retrograde and anterograde directions [53–56]. Entry of proteins from the ER continues however, and therefore proteins that cycle between the ER and Golgi accumulate in the ERGIC at 15°C [53]. When cells were incubated for 3 hours at 15°C, the localisation of ERGIC53 was altered from the large perinuclear stacks observed at 37°C (Fig 2C–2E, top panels), to smaller, more numerous compartments with a more disperse distribution (Fig 2C–2E, bottom panels), consistent with accumulation of ERGIC53 in ERGIC compartments [53, 57]. At 37°C, a proportion of both CD8^TMD*^ and CD8^*PMCA*^ co-localised with ERGIC53 (Fig 2C and 2D, top panels). After 3 hours at 15°C however, a marked redistribution of CD8^TMD*^ and CD8^*PMCA*^ occurred, with a substantially greater proportion of each chimera being concentrated in ERGIC53-positive compartments (Fig 2C and 2D, bottom panels). We considered the possibility that accumulation of CD8^TMD*^ and CD8^PMCA*^ in the ERGIC might be due to inhibition of anterograde transport of newly synthesised protein from the ERGIC to the cis-Golgi. However, CD8^*WT*^ was not observed in ERGIC53-positive compartments after 3 hours at 15°C (Fig 2E, bottom panel). This suggests that the accumulation of CD8^TMD*^ and CD8^*PMCA*^ in the ERGIC at 15°C was not solely due to inhibition of anterograde transport from the ERGIC (which would also cause accumulation of CD8^*WT*^), but also reflects inhibition of retrograde transport that returns CD8^TMD*^ and CD8^*PMCA*^, but not CD8^*WT*^, from the ERGIC to the ER. Thus, on the basis of these results, we conclude that retrieval mechanisms contribute to the ER localisation of CD8 containing non-native TMDs.

### Rer1 contributes to the retrieval of CD8^TMD*^

**Fig 3:**
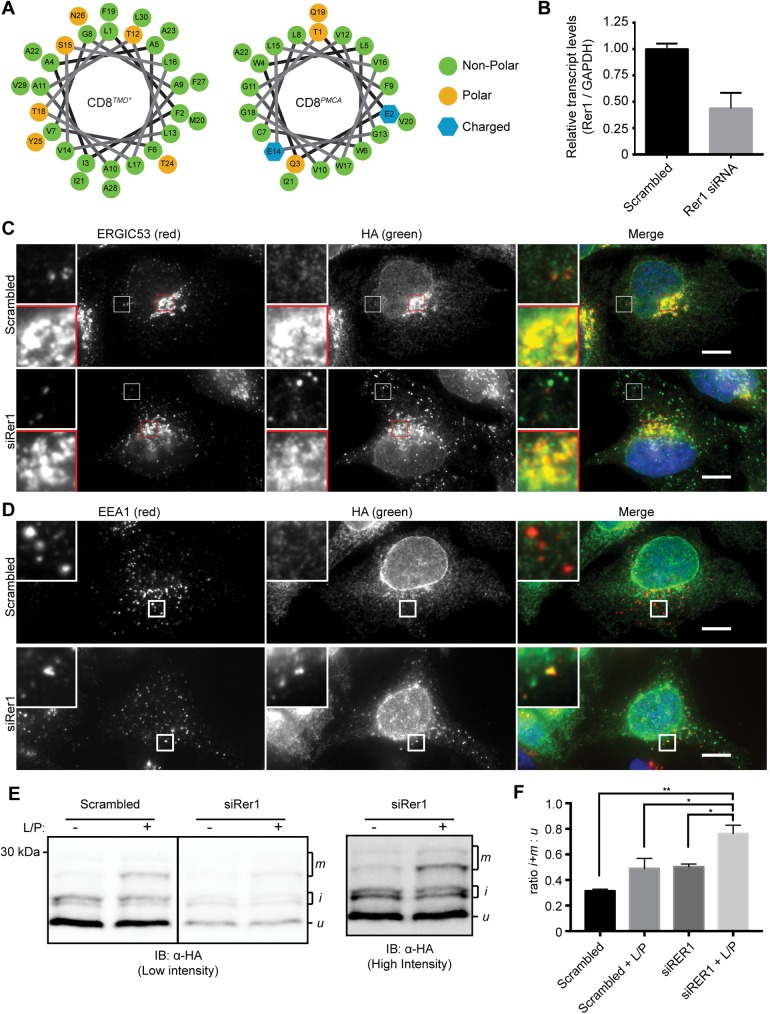
CD8^TMD*^ retrieval from the Golgi is mediated by Rer1. (A) Helical wheel projections for the transmembrane domains of CD8^TMD*^ and CD8^*PMCA*^. Numbering starts from the N-terminus of the predicted TMD. (B) Rer1 mRNA levels were determined by qPCR 72h post transfection with scrambled or Rer1 siRNA and normalised relative to GAPDH mRNA levels. (C and D) Cells expressing CD8^TMD*^ were transfected with scrambled siRNA or siRNA targeting Rer1 as indicated. 72 hours subsequently, cells were fixed and co-immunostained with antibodies against (C) ERGIC53 and HA or (D) EEA1 and HA. Cell nuclei were stained with DAPI. Scale bars indicate 10 μm. (E) Cell expressing CD8^TMD*^ were transfected with scrambled siRNA or siRNA targeting Rer1 for 72 hours. Immediately prior to harvesting, cells were incubated with leupeptin and pepstatin A (L/P) for 5 hours or left uninhibited. Whole cell lysates were separated by SDS-PAGE and analysed by immunoblotting with antibodies against HA. (F) Signal intensities from high molecular weight ‘i’ and ‘m’ glycoforms expressed as a ratio to the unprocessed ‘u’ glycoform. Scrambled siRNA vs RER1 siRNA + L/P, p = 0.0015; Rer1 siRNA vs Rer1 siRNA + L/P, p = 0.0267; scrambled siRNA + L/P vs Rer1 siRNA + L/P, p = 0.0337; one way ANOVA with Tukey’s multiple comparisons test. Data represents mean ±S.E.M. from 3 independent experiments.

We next addressed the mechanism that mediates retrieval of CD8 TMD chimeras from the Golgi. Rer1 is a membrane protein that cycles between the ER and Golgi and has been implicated in the retrieval of proteins from the Golgi to the ER via recognition of exposed polar residues within TMDs. Helical wheel projections of the TMDs of CD8^TMD*^ and CD8^*PMCA*^ indicated that the polar and charged residues are spatially clustered in both proteins, forming potential Rer1 interaction motifs [30]. This is particularly true of CD8^TMD*^, where 5 polar residues were predicted to be grouped on one side of the helix (Fig 3A). In order to determine whether Rer1 contributes to the ER localisation of CD8^TMD*^, we examined the effect of siRNA-mediated knockdown of Rer1. Rer1 depletion (~57% reduction in Rer1 mRNA levels, Fig 3B; > 80% reduction in levels of exogenously expressed Rer1-V5 protein, S4A Fig) caused a marked change in the distribution of CD8^TMD*^, such that it appeared predominantly in punctate structures that were distributed throughout the cell (Fig 3C, compare top and bottom). A similar effect was observed using a second independent siRNA oligonucleotide targeting Rer1 (S4B Fig). The puncta induced by Rer1 siRNA did not colocalise with ERGIC53 (Fig 3C, bottom, white inset box), indicating that they do not correspond to the ERGIC or cis-Golgi. Rer1 knockdown has recently been shown to cause a misfolded membrane protein usually degraded by ERAD to accumulate in lysosomes [36], and we therefore examined whether these puncta represented CD8^TMD*^ *en route* to lysosomes. Indeed, some co-localisation between CD8^TMD*^-containing puncta and the endosomal marker EEA1 was observed upon Rer1 depletion (Fig 3D, bottom), but not in scrambled siRNA-treated cells (Fig 3D, top). Notably, only a proportion of the CD8^TMD*^ positive puncta were also positive for EEA1, consistent with CD8^TMD*^ transiting through endosomes in Rer1 depleted cells. The appearance of CD8^TMD*^ in EEA1 positive structures upon Rer1 knockdown indicates that protein which escapes retrieval from the Golgi is ultimately sorted to lysosomes for degradation. A small increase in the proportion of CD8^TMD*^ was seen in the Golgi modified ‘i’ and ‘m’ forms in Rer1 depleted cells than in control cells (Fig 3E), consistent with a greater proportion of CD8^TMD*^ reaching the Golgi in the absence of Rer1-mediated retrieval. Lysosomal protease inhibitors stabilised the higher molecular weight ‘m’ glycoform generated in the trans-Golgi network [43], and this effect was most apparent in Rer1 depleted cells (Fig 3E and 3F). Hence following treatment with leupeptin and pepstatin, the ratio of Golgi-modified ‘i’ and ‘m’ glycoforms relative to the ER retained ‘u’ form was significanly (P = 0.0337) higher in Rer1 depleted cells than in cells treated with scrambled siRNA (Fig 3F), suggesting a greater proportion of CD8^TMD*^ escaped the ER in the absence of Rer1. Together these results provide evidence that Rer1-mediated retrieval contributes to maintaining ER localisation of CD8^TMD*^, and that CD8^TMD*^ which escapes retrieval is at least in part targeted to lysosomes for degradation.

### A Golgi quality control checkpoint for transmembrane domains

**Fig 4:**
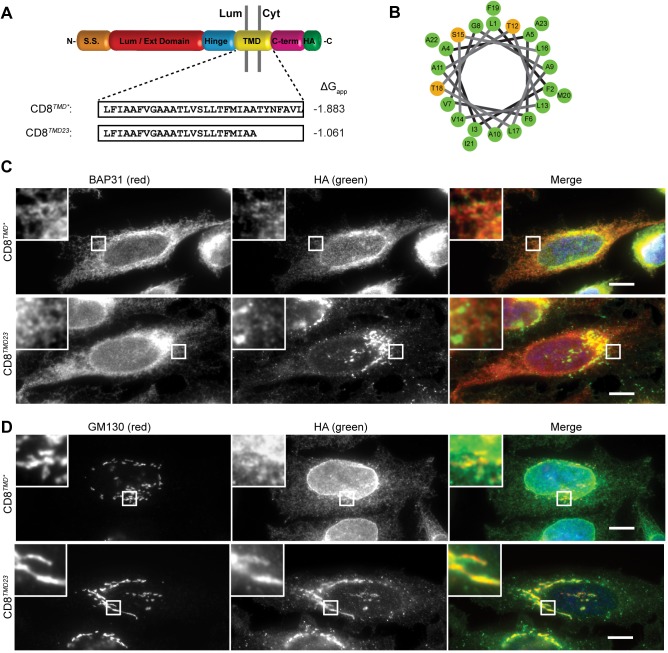
CD8^*TMD23*^ is a substrate from Golgi quality control. (A) Cartoon schematic of the domain structure of CD8α. The amino acid compositions and predicted ΔG_app_ for TMD membrane insertion of CD8^TMD*^ and CD8^*TMD23*^ are shown. (B) Helical wheel projection for the transmembrane domain of CD8^*TMD23*^. Numbering starts from the N-terminus of the predicted TMD. (C and D) Cells expressing CD8^TMD*^ or CD8^*TMD23*^ were fixed and co-immunostained with antibodies against (C) BAP31 or (D) GM130 as well as the epitope tag HA in all cases. Cell nuclei were stained with DAPI. Scale bars indicate 10 μm.

**Fig 5:**
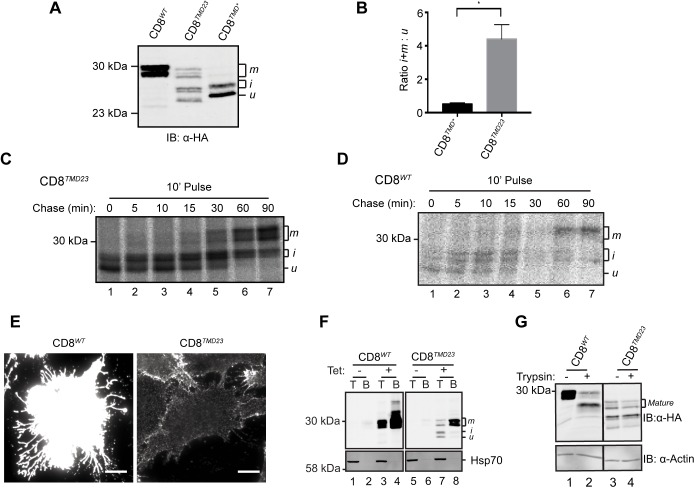
CD8^*TMD23*^ is predominantly retained in the Golgi. (A) Whole cell lysates of cells expressing CD8^*WT*^, CD8^TMD*^ or CD8^*TMD23*^ were separated by SDS-PAGE and analysed by immunoblotting with antibodies against HA. (B) Ratios of signal intensities of intermediate ‘i’ and mature ‘m’ to unprocessed ‘u’ glycoforms of CD8^TMD*^ or CD8^*TMD23*^ at steady state (n = 3). P = 0.0109, unpaired t-test. (C & D) Cells expressing (C) CD8^*TMD23*^ or (D) CD8^*WT*^ were pulse labelled with [^35^S] met/cys for 10 minutes and chased for up to 90 minutes as indicated. Samples were immunoprecipitated with antibodies against HA, separated by SDS-PAGE and analysed by phosphorimaging. (E) In parallel, cells expressing CD8^*WT*^ or CD8^*TMD23*^ were chilled on ice and labelled with antibodies against the extracellular domain of CD8 prior to fixation in formaldehyde. Note that pictures were taken in parallel with equal exposure times. Scale bars indicate 10 μm. (F) Cells expressing CD8^*WT*^ or CD8^*TMD23*^ were chilled on iced water and labelled with biotin prior to cell lysis. Total cell lysates samples were taken and the remaining cell lysate incubated with neutravidin to isolate biotinylated (cell surface) protein. Total ‘T’ and biotinylated ‘B’ samples were separated by SDS-PAGE and analysed by immunoblotting with antibodies against HA, tubulin and Hsp70. (G) Cells expressing CD8^*WT*^ or CD8^*TMD23*^ were incubated on ice and treated with trypsin. Subsequently cells were washed and whole cell lysates separated by SDS-PAGE and analysed by immunoblotting with antibodies against HA and actin.

We next investigated which features of the transmembrane sequence in CD8^TMD*^ contributed to its ER localisation. At 30 residues, the transmembrane sequence in CD8^TMD*^ is likely to be longer than the thickness of the membrane, a so-called positive mismatch [58]. We therefore examined the effect of shortening the TMD of CD8^TMD*^ by removing the seven C-terminal residues of the TMD to create CD8^*TMD23*^, and in doing so also removed three of the polar amino acids that contributed to the putative Rer1 recognition motif (Fig 4A and 4B). These changes markedly altered the subcellular distribution of the chimera, and in contrast to the perinuclear and reticular localisation of CD8^TMD*^, CD8^*TMD23*^ was predominantly localised to a ribbon-like structure typical of the mammalian Golgi apparatus (Fig 4C, middle panels). Relatively little CD8^*TMD23*^ appeared to be ER retained, as judged by co-localisation with BAP31 (Fig 4C). A much higher degree of co-localisation with the cis-Golgi marker GM130 was observed (Fig 4D), suggesting that more CD8^*TMD23*^ escaped from ER retention and / or retrieval mechanisms. Consistent with this observation, a significantly greater proportion of CD8^*TMD23*^ was observed in higher molecular weight Golgi modified ‘i’ and ‘m’ glycoforms than CD8^TMD*^ at steady state (Fig 5A and 5B; P = 0.0109). To examine ER-to-Golgi transport of CD8^*TMD23*^ more directly, we followed the acquisition of Golgi modifications using pulse-chase radiolabelling as described previously. Like CD8^TMD*^ (Fig 2A), CD8^*TMD23*^ was predominantly synthesised as the ‘u’ form with a smaller amount of the glycosylated intermediate ‘i’ during the 10 minute pulse (Fig 5C, lane 1). The mature glycoform ‘m’ appeared after 15–30 minutes of chase (Fig 5C lane 4) as observed for CD8^TMD*^ (Fig 2A). However, in contrast to CD8^TMD*^ (Fig 2A), essentially all of the CD8^*TMD23*^ was modified to the ‘i’ and ‘m’ forms during the timecourse of the experiment (Fig 5C). As observed for CD8^*WT*^ (Fig 5D), very little, if any, CD8^*TMD23*^ remained in the ‘u’ precursor form after 60 minutes of chase (Fig 5C, lane 6). The lack of modification of CD8^TMD*^ (Fig 2A) was not due to its luminal domain being resistant to O-glycosylation, since treatment of cells with brefeldin A to redistribute Golgi glycosidase enzymes to the ER, rapidly converted all of the CD8^TMD*^ to the higher molecular weight ‘i’ and ‘m’ forms [43]. These data suggest that like CD8^*WT*^, the great majority of CD8^*TMD23*^ escaped from the ER and reached the Golgi within 70 minutes (10 minutes pulse plus 60 minutes chase).

Thus, we conclude that the seven residues in the C-terminal portion of the TMD were primarily responsible for the ER retention / retrieval of CD8^TMD*^. The effect of deleting these residues could reflect disruption of the putative Rer1 recognition motif and / or the effect of shortening the TMD and thus reducing a hydrophobic mismatch. However, it is noteworthy that the localisation of CD8^*TMD23*^ was distinct from that of CD8^TMD*^ upon treatment with Rer1 siRNA (compare Figs 4D and 3C respectively). This observation indicates that the two TMDs are handled differently upon arrival at the Golgi. Hence, whilst CD8^TMD*^ appeared to be targeted to the endo/lysosomal system upon Rer1 siRNA, CD8^*TMD23*^ was predominantly retained at the Golgi.

Although CD8^*TMD23*^ was efficiently transported to the Golgi, little was seen at the plasma membrane of permeabilised cells. Cell surface labelling of intact cells with an antibody against the extracellular domain of CD8 revealed only faint cell surface labelling in cells expressing CD8^*TMD23*^ compared to that seen in cells expressing CD8^*WT*^ (Fig 5E). Similarly, treatment with membrane-impermeable Sulfo-NHS-SS-Biotin to selectively label cell surface protein resulted in modification of only a small amount of CD8^*TMD23*^ compared to CD8^*WT*^ (Fig 5F, compare lanes 8 and 4). Whilst treatment of intact of cells with trypsin resulted in proteolysis of the majority of CD8^*WT*^ (Fig 5G, lane 2), CD8^*TMD23*^ was largely protected from externally added protease (Fig 5G, lane 4). Together, these experiments show that relatively little CD8^*TMD23*^ was located at the cell surface. Thus, even though CD8^*TMD23*^ was able to evade ER retention and / or retrieval mechanisms, it was subsequently retained at the Golgi apparatus (Fig 4D). These findings are important as they suggest the existence of a quality control checkpoint at the Golgi which is able to recognise features of the non-native TMD sequence present in CD8^*TMD23*^.

**Fig 6:**
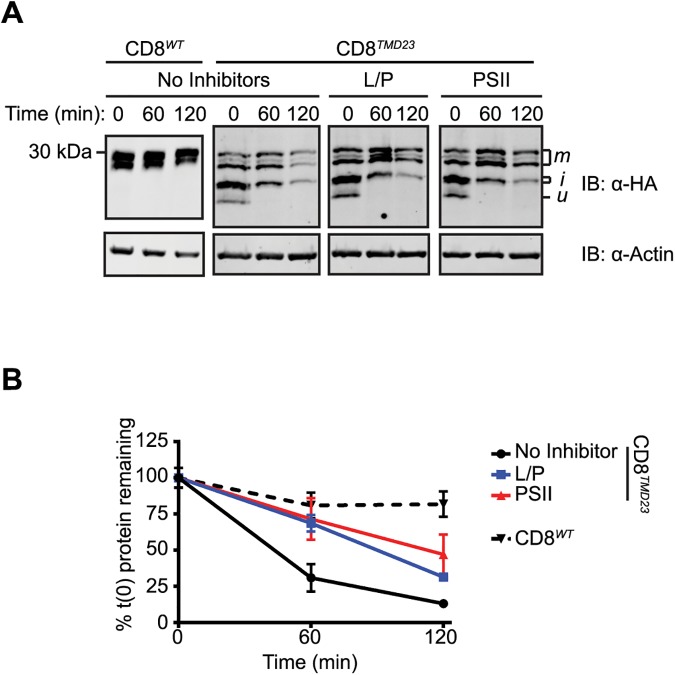
CD8^T*MD23*^ is degraded by proteasomes and lysosomes. (A) Cells expressing CD8^*WT*^ or CD8^*TMD23*^ were treated with cycloheximide (CHX) to prevent further protein synthesis. Whole cell lysates were harvested at the indicated chase time, separated by SDS-PAGE and analysed by immunoblotting with antibodies against HA and the loading control actin. Where indicated, cells expressing CD8^*TMD23*^ were treated with leupeptin and pepstatin A (LP) or PSII concurrently with CHX. (B) Signal intensities from (A) were quantified, normalised relative to the loading control and expressed as a percentage of the protein level at the start of the chase. Data represents mean ±S.E.M. from 3 independent experiments.

Little is known about Golgi quality control mechanisms, particularly in mammalian cells, and therefore, we next examined the fate of CD8^*TMD23*^. The stability of CD8^*TMD23*^ was analysed by cycloheximide chase assays in which cells expressing CD8^*TMD23*^ were treated with cycloheximide to inhibit further protein synthesis, and then ‘chased’ for up to 120 minutes in the presence of cycloheximide. Cells were harvested at different times following the addition of cycloheximide, and the amount of CD8^*TMD23*^ remaining at each time point was determined by SDS-PAGE and immunoblotting. In contrast to CD8^*WT*^, the levels of which remained relatively constant, CD8^*TMD23*^ was rapidly lost during the 120 minute chase (Fig 6A), suggesting that CD8^*TMD23*^ was unstable. Inclusion of the proteasome inhibitor Z-LLF-CHO (PSII) in the chase media substantially slowed the loss of CD8^*TMD23*^, with twice as much protein remaining at each time point (Fig 6A and 6B). Since it is not clear how protein would be targeted to proteasomes directly from the Golgi, this observation suggests that some CD8^*TMD23*^ is recognised at the ER and degraded via ERAD, as previously demonstrated for CD8^TMD*^ [43]. However, in contrast to CD8^TMD*^, proteasome inhibition did not lead to accumulation of the ‘u’ precursor in the ER (Fig 6A), indicating that any CD8^*TMD23*^ not degraded by ERAD was instead transported to the Golgi and underwent post translational modification to the ‘i’ and ‘m’ forms (Fig 6A). Treatment with the lysosomal protease inhibitors leupeptin and pepstatin A also reduced CD8^*TMD23*^ degradation (Fig 6A and 6B), suggesting that CD8^*TMD23*^ retained at the Golgi is targeted to lysosomes for degradation.

**Fig 7:**
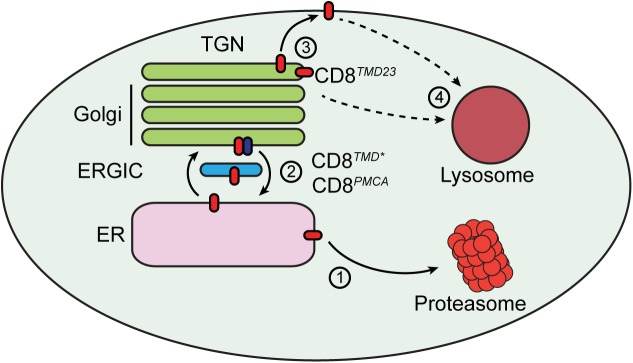
Transmembrane domain quality control at the ER and Golgi. A model for the handling of proteins containing misassembled transmembrane domains (red ovals). (1) Proteins containing misassembled transmembrane domains may be retained in the ER and degraded via ERAD. (2) A proportion of these proteins may cycle between the ER and Golgi. Golgi-to-ER retrieval is at least in part mediated by Rer1 (blue oval). (3) Proteins that are not retrieved to the ER may be retained by Golgi quality control machinery. (4) Proteins that escape ER retrieval are degraded in lysosomes. Some protein may traffic to the cell surface. It is not clear whether trafficking to the plasma membrane is a prerequisite for targeting to lysosomes, or whether proteins containing aberrant TMDs can be directly targeted to the endo/lysosomal system from the Golgi.

Together, these results suggest a model whereby proteins with non-native TMDs are able to exit the ER and travel to the Golgi apparatus, from where they can either be returned to the ER via retrograde transport involving Rer1, or are retained via an unknown mechanism and ultimately routed to lysosomes for degradation (Fig 7).

## Discussion

The role of TMD-based signals in the quality control of misfolded membrane proteins in mammalian cells is poorly understood. This has remained a difficult question to address since few model proteins that contain aberrant transmembrane segments but folded luminal and cytoplasmic domains have been characterised. In this study, we have used TMD chimeras based on CD8 to provide a simple model system to study how misassembled TMDs are identified and handled in the secretory pathway. Our results suggest that non-native TMDs are recognised by multiple checkpoints in the secretory pathway (Fig 7). At the level of the ER, we found that both retention and retrieval facilitate ER localisation of CD8 chimeras containing non-native TMDs, whilst additional mechanism(s) at the Golgi retain proteins that escape ER retention/retrieval.

Both CD8^TMD*^ and CD8^*PMCA*^ were localised to the ER, and since substitution of the endogenous TMD of CD8 does not prevent folding of the ER luminal / extracellular domain ([43] and refs therein) or integration into the ER membrane, we conclude that specific features of the non-native TMD sequences prevent anterograde trafficking of these chimeras to the cell surface. The chimeras are degraded via a proteasome-dependent pathway [43], suggesting that the ERAD machinery recognises signals embedded within the lipid bilayer to promote degradation of proteins containing non-native TMDs (Fig 7, step 1). The properties of the two exogenous TMD sequences in CD8^TMD*^ and CD8^*PMCA*^ are quite distinct in terms of their amino acid composition, hydrophobicity and length (Fig 1A, S1A Fig), showing that diverse TMD sequences have the potential to mediate ER retention and degradation. However, both sequences do contain a number of polar residues that could act as determinants for recognition by transmembrane proteins that monitor TMD assembly. Quality control factors that could potentially contribute to ER retention of CD8^TMD*^ and CD8^*PMCA*^ include calnexin, which has been implicated in the retention of several proteins containing misassembled TMDs [14–17] and ERAD E3 ubiquitin ligases such as Hrd1, which in *S*. *cerevisiae* recognise aberrant TMDs directly [18, 19]. An additional possibility to the recognition of specific sequence motifs is that partitioning into particular membrane environments such as lipid microdomains or lipid droplets due to the physiochemical properties of the TMD sequences contributes to quality control of proteins containing aberrant TMDs [24]. In this regard it is noteworthy that bioinformatic and experimental studies have provided evidence that TMD length is an important determinant of membrane protein localisation, with longer more hydrophobic TMDs being located at the cell surface and shorter more hydrophilic TMDs favouring ER/Golgi localisation [10]. A discrepancy between the length of the TMD and the thickness of the membrane, such is the case for CD8^TMD*^, is predicted to be energetically unfavourable and may lead to tilting within the membrane in order to minimise the energetic cost of hydrating hydrophobic regions [59]. Whether such alterations in the lipid bilayer contribute to the quality control of proteins possessing aberrant TMDs is an important and as yet unanswered question.

Despite their predominant ER localisation and degradation via ERAD, neither CD8^TMD*^ nor CD8^*PMCA*^ were stringently retained in the ER. A considerable pool of each protein escaped to the Golgi apparatus and underwent retrieval via retrograde transport through the ERGIC (Fig 7, step 2). This is interesting as it indicates that retention of non-native TMDs by ER quality control may be relatively inefficient, and depend upon retrieval from the Golgi to maintain ER localisation and promote ERAD. Indeed, knockdown of Rer1, which has been previously been implicated in the retrieval of ER resident and misfolded / misassembled membrane proteins [14, 30, 36, 60, 61], increased escape of CD8^TMD*^ from the ER and lead to its accumulation in endo/lysosomal compartments, suggesting that lack of retrieval from the Golgi results in delivery to lysosomes (Fig 7, step 4). Previous studies have identified polar residues within TMDs as being critical to recognition by Rer1 [28, 30, 34], and both CD8^TMD*^ and CD8^*PMCA*^ contain clusters of polar residues that could potentially form Rer1 binding motifs (Fig 3A). Deletion of the seven residues at the cytoplasmic C-terminal end of the TMD of CD8^TMD*^, which contained three polar residues, caused the resulting protein CD8^*TMD23*^ to localise to the Golgi. The simplest interpretation of this observation is that the deletion disrupted the putative Rer1 recognition motif, and thus prevented retrieval from the Golgi to the ER, leading to accumulation in the Golgi. However, deletion of these residues also shortened the length of the predicted TMD in CD8^*TMD23*^ and therefore the difference in length may also contribute to the distinct localisation of CD8^TMD*^ and CD8^*TMD23*^. Indeed, shortening the TMD also appeared to reduce static retention of CD8^*TMD23*^ in the ER, since essentially all pulse-labelled CD8^*TMD23*^ received Golgi modifications within 60 min, in contrast to only around 50% of CD8^TMD*^ [43]. In either case, the observation that CD8^*TMD23*^ accumulates in the Golgi suggests that a second TMD-based checkpoint operates to retain CD8^*TMD23*^ at this compartment (Fig 7, step 3).

While CD8^*TMD23*^ appeared to localise predominantly to the Golgi at steady state, this chimera was degraded by a combination of both proteasome and lysosome dependent pathways. Proteasome inhibition lead to accumulation of the higher molecular weight glycoforms but not the unmodified precursor, suggesting that CD8^*TMD23*^ not degraded by ERAD exits the ER and moves to the Golgi as observed for several other misfolded proteins [62, 63]. This would be consistent with a model whereby static retention of CD8^*TMD23*^ in the ER is inefficient, with any protein not degraded by ERAD being exported to the Golgi apparatus. From the Golgi, CD8^*TMD23*^ could be delivered to lysosomes for degradation (Fig 7, step 4) directly as has been observed in yeast for proteins containing exposed polar residues [40, 42]. It is also possible that trafficking of CD8^*TMD23*^ from the Golgi to the lysosome may occur via the cell surface (Fig 7, step 4). In this scenario, CD8^*TMD23*^ would move from the Golgi to the cell surface, from where it would be incorporated into endocytic vesicles and subsequently sorted for deliver to lysosomes. Further work will be aimed at elucidating the contribution of endocytosis to the handling of proteins containing unassembled TMDs.

Interestingly, the distribution of CD8^*TMD23*^, which was localised to Golgi stacks, differed from that of CD8^TMD*^ upon Rer1 siRNA treatment, which appeared in punctate structures. The reasons for this have not been defined, but may reflect different handling of CD8^TMD*^ and CD8^*TMD23*^ in the Golgi. It is conceivable that CD8^TMD*^ is more efficiently targeted to the endo/lysosomal system than CD8^*TMD23*^, and therefore localised with greater frequency in punctate structures. Indeed, polar residues within TMDs have previously been implicated in protein sorting from the Golgi to vacuoles in *S*. *cerevisiae* [37, 40]. Whether polar residues are similarly crucial to TMD recognition in the Golgi of mammalian cells requires further examination, and future work will be focussed on defining quality control at the Golgi.

## Supporting information

S1 Fig Cartoon diagram showing the transmembrane domains sequences (bold, in boxes) from the constructs used in this study. (TIF)

S2 Fig Antibody labelling indicates CD8^*SERCA*^ does not integrate into ER membranes. (A) HeLa cells were fixed and permeabilised with Triton X-100 or digitonin, then co-immunostained with antibodies against a cytoplasmic epitope of the ER membrane protein BAP31 and the luminal ER protein calreticulin (CRT). Cell nuclei were stained with DAPI (represented in blue in merged images). Scale bars represent 10 μm. (B) Cells expressing CD8^TMD*^ were fixed and permeabilised with Triton X-100 or digitonin. Cells were co-immunostained with antibodies against the luminal domain of CD8 or the cytosolic HA tag. (C) Cells expressing CD8^*PMCA*^ were treated as in (B). (D) Cells expressing CD8^*SERCA*^ were treated as in (B).
(TIF)

S3 Fig Carbonate extraction of CD8^*TMD23*^, CD8^*PMCA*^ and CD8^*SERCA*^. Cells were induced to express (A) CD8^*TMD23*^, (B) CD8^*PMCA*^ or (C) CD8^*SERCA*^. Lysates were subjected to alkaline extraction as described previously (Briant et al, 2015, J Cell Sci 128(22):4112–25). Samples of the input postnuclear supernatant (PNS), cytosol, total membranes, carbonate extracted material (CO3 supernatant) and carbonate resistant material (CO3 pellet) were analysed by reducing SDS-PAGE and immunoblotting with antibodies to CD8 (anti-HA), an ER luminal protein (BiP) and an integral ER membrane protein (BAP31). The efficiency of the carbonate extraction is shown by the enrichment of BiP in the CO3 supernatant and BAP31 in the CO3 pellet.
(TIF)

S4 Fig Rer1 mediates CD8^TMD*^ retrieval from the Golgi. (A) HEK293 cells stably expressing V5-tagged Rer1 were left untreated, treated with siRNA targeting Rer1 or treated with a control siRNA targeting calnexin. 72h post transfection, whole cell lysates were taken and knockdown efficiency assessed by immunoblotting with antibodies against the V5 tag and Rer1. (B) HeLa cells expressing CD8^TMD*^ were treated with scrambled siRNA or second siRNA targeting Rer1. The distribution of CD8TMD* was subsequently detected with α-HA antibodies. Scale bars indicate 10 μm.
(TIF)
