# Supplementary figures and images for: Transmembrane domain quality control systems operate at the endoplasmic reticulum and Golgi apparatus
Source: PLoS One. 2017 Apr 6;12(4):e0173924. doi: 10.1371/journal.pone.0173924 (PMC5383021; doi:10.1371/journal.pone.0173924)

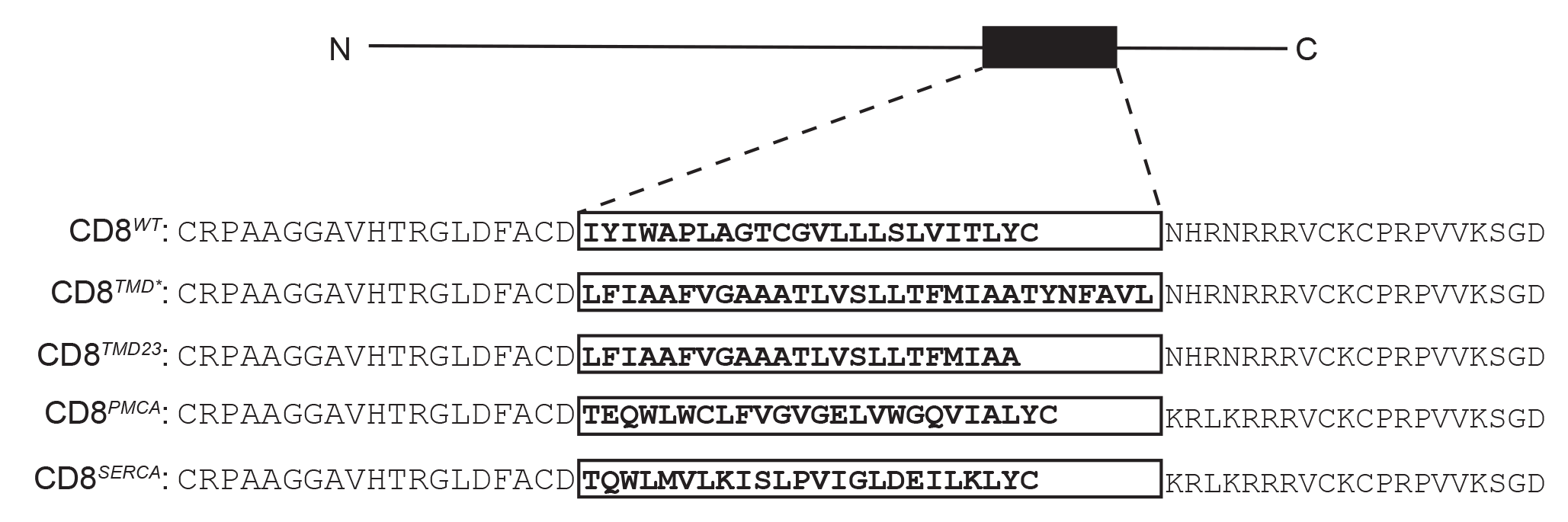

Supplement: S1 Fig — (TIF) [file pone.0173924.s001.tif]

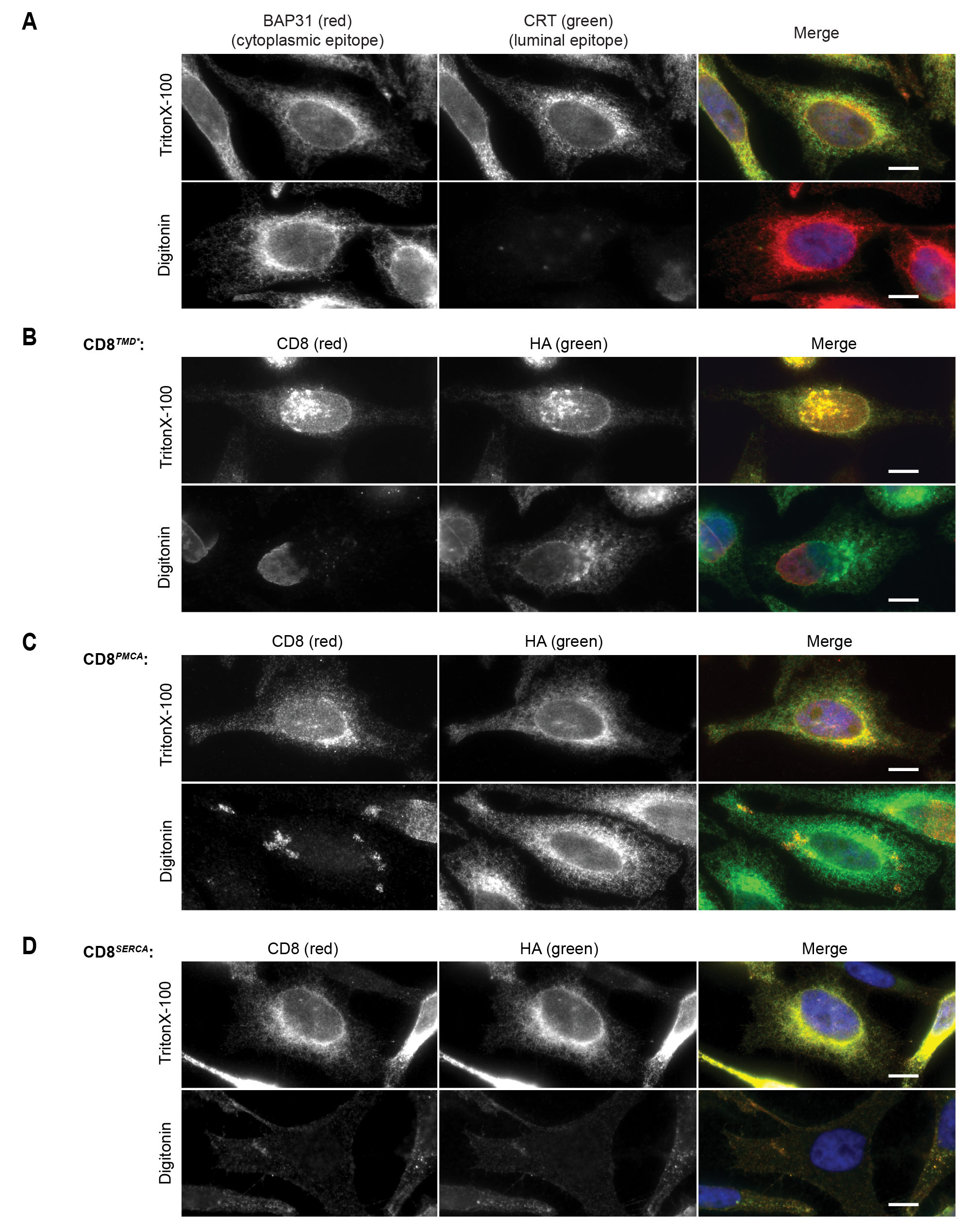

Supplement: S2 Fig — (A) HeLa cells were fixed and permeabilised with Triton X-100 or digitonin, then co-immunostained with antibodies against a cytoplasmic epitope of the ER membrane protein BAP31 and the luminal ER protein calreticulin (CRT). Cell nuclei were stained with DAPI (represented in blue in merged images). Scale bars represent 10 μm. (B) Cells expressing CD8TMD* were fixed and permeabilised with Triton X-100 or digitonin. Cells were co-immunostained with antibodies against the luminal domain of CD8 or the cytosolic HA tag. (C) Cells expressing CD8PMCA were treated as in (B). (D) Cells expressing CD8SERCA were treated as in (B). (TIF) [file pone.0173924.s002.tif]

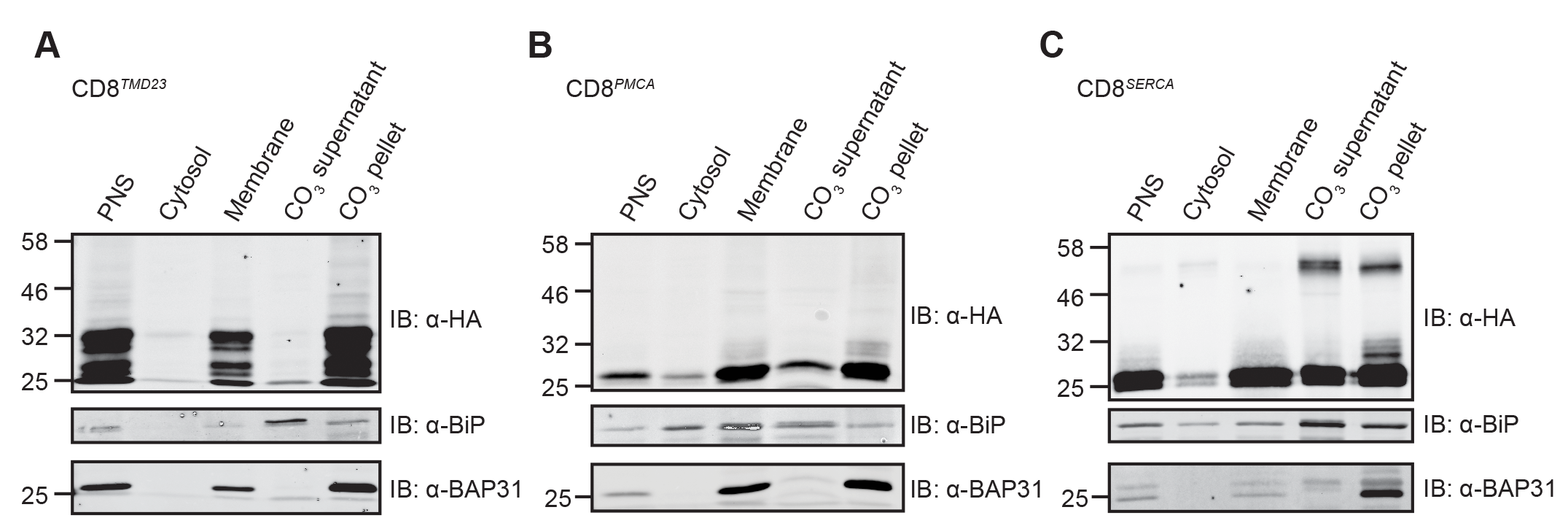

Supplement: S3 Fig — Cells were induced to express (A) CD8TMD23, (B) CD8PMCA or (C) CD8SERCA. Lysates were subjected to alkaline extraction as described previously (Briant et al, 2015, J Cell Sci 128(22):4112–25). Samples of the input postnuclear supernatant (PNS), cytosol, total membranes, carbonate extracted material (CO3 supernatant) and carbonate resistant material (CO3 pellet) were analysed by reducing SDS-PAGE and immunoblotting with antibodies to CD8 (anti-HA), an ER luminal protein (BiP) and an integral ER membrane protein (BAP31). The efficiency of the carbonate extraction is shown by the enrichment of BiP in the CO3 supernatant and BAP31 in the CO3 pellet. (TIF) [file pone.0173924.s003.tif]

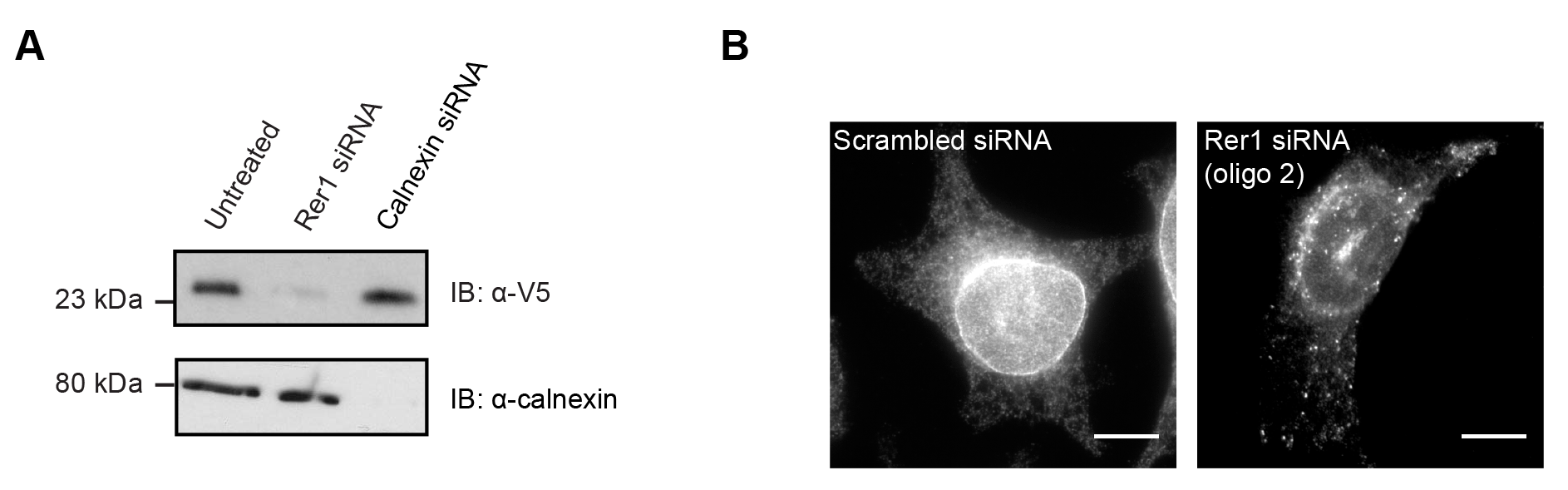

Supplement: S4 Fig — (A) HEK293 cells stably expressing V5-tagged Rer1 were left untreated, treated with siRNA targeting Rer1 or treated with a control siRNA targeting calnexin. 72h post transfection, whole cell lysates were taken and knockdown efficiency assessed by immunoblotting with antibodies against the V5 tag and Rer1. (B) HeLa cells expressing CD8TMD* were treated with scrambled siRNA or second siRNA targeting Rer1. The distribution of CD8TMD* was subsequently detected with α-HA antibodies. Scale bars indicate 10 μm. (TIF) [file pone.0173924.s004.tif]
